# The Reactivity-Enhancing Role of Water Clusters in Ammonia Aqueous Solutions

**DOI:** 10.1021/acs.jpclett.3c01810

**Published:** 2023-08-25

**Authors:** Giuseppe Cassone, Franz Saija, Jiri Sponer, Sason Shaik

**Affiliations:** † Institute for Physical-Chemical Processes, Italian National Research Council (CNR-IPCF), Viale Stagno d’Alcontres 37, 98158 Messina, Italy; ‡ Institute of Biophysics of the Czech Academy of Sciences, Královopolská 135, 61265 Brno, Czechia; ¶ Institute of Chemistry, The Hebrew University of Jerusalem, Edmond J. Safra Campus, Givat Ram, Jerusalem 9190401, Israel

## Abstract

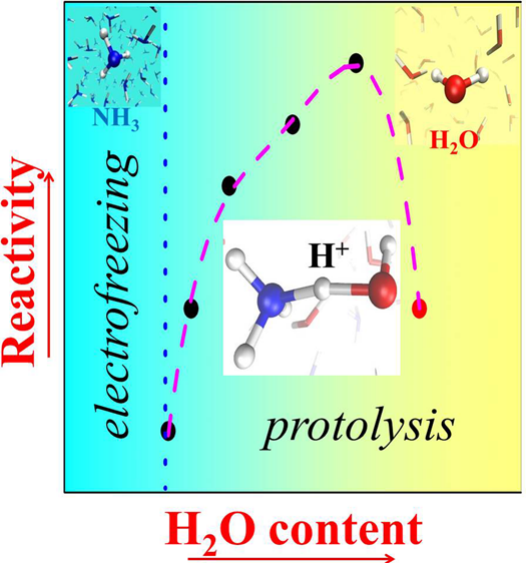

Among the many prototypical acid–base systems, ammonia aqueous solutions hold a privileged place, owing to their omnipresence in various planets and their universal solvent character. Although the theoretical optimal water–ammonia molar ratio to form NH_4_^+^ and OH^–^ ion pairs is 50:50, our *ab initio* molecular dynamics simulations show that the tendency of forming these ionic species is inversely (directly) proportional to the amount of ammonia (water) in ammonia aqueous solutions, up to a water–ammonia molar ratio of ∼75:25. Here we prove that the reactivity of these liquid mixtures is rooted in peculiar microscopic patterns emerging at the H-bonding scale, where the highly orchestrated motion of 5 solvating molecules modulates proton transfer events through local electric fields. This study demonstrates that the reaction of water with NH_3_ is catalyzed by a small cluster of water molecules, in which an H atom possesses a high local electric field, much like the effect observed in catalysis by water droplets [PNAS2023, 120, e23012061203703696810.1073/pnas.2301206120PMC10120050].

Concepts like acid, base, and reactivity constitute the foundation of chemistry, as they are essential in understanding how molecules interact with one another in a variety of phenomena. The capability of transferring protons H^+^ from a molecular species to another one is, indeed, at the heart of a plethora of processes which occupy a key place in environmental, industrial, analytical, and medicinal (bio)chemistry.^1−3^

Water–ammonia mixtures govern subtle equilibria in atmospheric processes.^4,5^ Besides, the behavior of the interiors of giant icy planets such as Uranus and Neptune—but also of icy bodies in the outer Solar System and the formation of planets itself—depends on the peculiar physical and chemical properties of water–ammonia mixtures.^6^ Additionally, understanding the physical and chemical properties of these mixtures can provide insight into the potential for life in these environments, as well as the plausibility of prebiotic molecules formation.^7^ It is not surprising, therefore, that among the many prototypical acid–base systems ammonia aqueous solutions hold a privileged place and that the reaction between ammonia and water is among the most used and effective textbook examples of acid and base concepts.

The simple presence of ammonia itself in water affects the acidity of the mixture, with the formation of ammonium ions NH_4_^+^ influencing the pH of the solution via the following nominal reaction
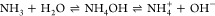
1This way, upon mixing, the reactivity of the system can be measured by the transformation of the liquid mixture to NH_4_^+^ and hydroxide ions OH^–^ as the result of proton transfer events. Nowadays, it is well established that protolysis in H-bonded liquids is mediated by large fluctuations of local electric fields on the order of ∼1 V/Å^8−10^ and that the application of external fields can affect their catalytic activity and selectivity.^11−14^ In fact, *ab initio* molecular dynamics (AIMD) simulations have shown that field strengths on the order of ∼0.3 V/Å are capable of disproportionating water molecules into oxonium (H_3_O^+^) and hydroxide ions,^9,12^ a field threshold in fairly good agreement with the available experimental results.^15,16^ Among other things, all these first-principles investigations correctly predicted some experimental findings proving that in aqueous systems electric fields generated by the solvent are on the order of ∼1 V/Å^17^ and that field strengths of ∼0.3 V/Å are responsible for the solvation state of the proton.^18^ Moreover, the electric field at the interface of water microdroplets falls in the same range of magnitude^19^ and is believed to be the main cause of the well-known catalytic role of microdroplets.^20,21^ The application of intense (∼0.1–1.0 V/Å) external electric fields on liquid systems can hence be exploited as a means for measuring its reactivity both from the spectroscopic signatures emerging from, e.g., the vibrational Stark effect within the namesake spectroscopy^22−25^ and from a direct monitoring of field-induced chemical reactions.^26−29^ The possibility of employing static fields as a probe for proton transfer reactivity is even more direct in H-bonded systems^13^ because of a tailored coupling between the magnitude of those fields with those naturally associated with H-bonds and intermolecular interactions, which fall on the order of ∼1 V/Å.^30,31^

Although the application of static electric fields as intense as ∼0.3–0.5 V/Å is generally responsible for a measurable increase of the reactivity in condensed-phase molecular systems such as liquid^9,12^ and solid^14,32^ water, alcohols,^33−35^ and heterogeneous mixtures,^36,37^ recent findings have shown that the reactivity of liquid ammonia is not susceptible to even stronger fields.^38^ In fact, a genuine physical response has been observed for this system, where the external field drives a structural transition from the liquid to a solid structure in a phenomenon known as electrofreezing.^38^ Thus, contrary to many other H-bonded systems, it has not been possible to find a finite electric field dissociation threshold since NH_3_ molecules remained intact even if subjected to very intense fields exposure.

Nonetheless, insertion of an amount of water corresponding to a total molar ratio lower than 1% in liquid ammonia awakens the system’s reactivity. In fact, upon applying progressively stronger static electric fields on a 0.8:99.2 water–ammonia mixture, protolysis is observed starting from a molecular dissociation threshold of 0.40 V/Å, as shown in Figure 1 and in Figure S5 of the Supporting Information. This way, the inclusion of small water amounts is capable of opening otherwise inaccessible reaction pathways in the presence of external electric fields. In other words, the catalytic role of water turns the *physical* response of neat liquid ammonia into a genuine *chemical* one. By increasing further the quantity of water to 10%, 25%, and 50% with respect to the total molecular content in the water–ammonia samples, a progressive reduction of the electric field dissociation threshold to 0.30, 0.20, and 0.15 V/Å is observed, respectively (Figure 1 and Figures S1–S3 of the Supporting Information). The largest decrement of the dissociation threshold, and hence the highest increment of the reactivity of the ammonia aqueous solution, is reached at the maximum relative amount of water explored here (i.e., 75%). In fact, under the 75:25 stoichiometric composition of the water–ammonia mixture, molecules are already dissociated by fields as intense as 0.10 V/Å, as displayed in Figure 1 and in Figure S4 of the Supporting Information. Albeit one might be tempted to conclude that the larger the amount of water the higher the reactivity of the mixture, it is noteworthy that this latter field strength is only one-third of the pure water ionization threshold (i.e., 0.10 V/Å *vis-à-vis* 0.30 V/Å^9,12,15,16^). This finding implies that there exists a natural minimum ionization threshold, to which corresponds a maximum in chemical reactivity, in ammonia aqueous solutions depending on the relative amount of dissolved water.

**Figure 1:**
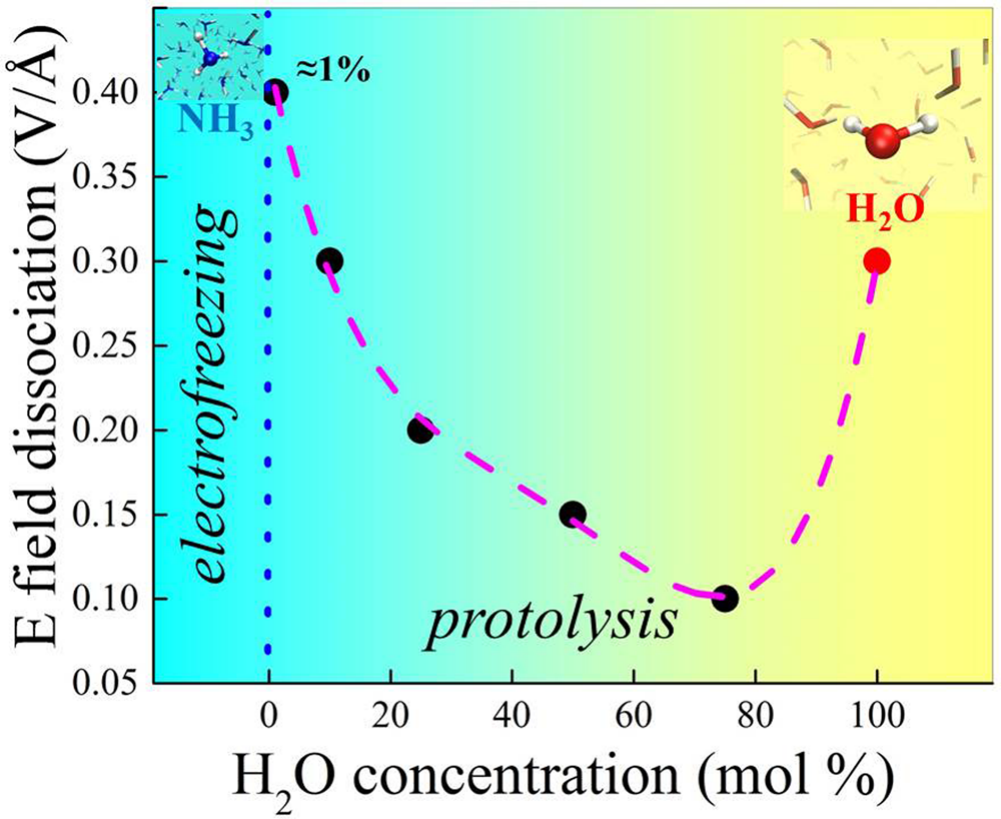
Black circles show the minimum electric field strength necessary to trigger successful dissociative events (i.e., protolysis) in ammonia aqueous solutions as a function of the molar concentration of water. In the left and right sides, the extreme cases corresponding to pure ammonia^38^ (no finite ionization threshold marked by the dotted blue asymptote) and neat water 0.30 V/Å^9,12,15,16^ (red circle), respectively. A fourth-order polynomial fit of the data points is shown as a guide for the eye (dashed magenta curve).

H-bonds energetics is very heterogeneous in water–ammonia mixtures under ambient conditions. In fact, whereas N–H···O and N–H···N bonds are quite weak and transient,^39,40^ O–H···O and O–H···N intermolecular bonds are strong and persistent.^41^ This means that the behavior of the protons H^+^ lying on these latter H-bonds should lead to dissociative events, with a particular preference for proton migrations originating from a H_2_O molecule toward a NH_3_ moiety. This is due to the higher proton affinity of NH_3_^42^ which, in turn, implies that the NH_4_^+^ cation may be found with larger probability than H_3_O^+^ in water–ammonia mixtures subjected to external electric fields. In fact, independently from the relative water–ammonia molar ratio, we have observed that the first electric-field-induced ionization events always involve HOH···NH_3_ H-bonded pairs and the transient formation of the well-known NH_4_OH molecular complex, in line with pioneering investigations on the behavior of the excess proton in ammonia aqueous solutions.^43^

The larger reactivity of the protons lying on the O–H···N bonds can be predicted *a priori* (i.e., at zero field and at standard neutral conditions) from the evaluation of a key indicator, known as the proton sharing coordinate δ. This parameter is capable of monitoring "proton excursion" events in the H-bond network and is defined as δ = *d*_OH_ – *d*_X__′__H_, where *d*_OH_ is the covalent bond length of a reference H_2_O molecule, whereas *d*_X__′__H_, with X = O, N, represents the length of the H-bond(s) that such a reference molecule donates—to either a nearby H_2_O or NH_3_ species—as depicted in the insets of Figure 2, both for the δ_O*w*O*w*_ (Figure 2a) and the δ_O*w*N_ case (Figure 2b). Of course, due to the fact that the H-bond(s) a molecule donates are longer than its own covalent bonds, δ generally assumes negative values. This way, the larger proneness toward dissociative events is witnessed by larger, less negative values of δ. As shown in Figure S6 of the Supporting Information, protons H^+^ shared in H-bonds between two H_2_O molecules sample distances which are measurably closer to the H-bond donor molecule than those explored by protons in HOH···NH_3_ H-bonded pairs. On these latter intermolecular bonds, indeed, positions occupied by H^+^ ions produce significantly larger values of the proton sharing coordinate δ_*OwN*_, independently from the relative amount of water in ammonia (Figure S6 of the Supporting Information). Such a finding quantitatively testifies that NH_3_ species are more prone to accept protons than H_2_O molecules. Notwithstanding the importance of discerning among the most reactive H-bonds, this finding does not provide any clue for interpreting the data presented in Figure 1 and indicates that there exists a precise balance in the amount of H_2_O and NH_3_ molecules in the mixture producing a condition of maximum reactivity. With the aim of shedding light on this aspect, it is worth investigating the behavior of protons for different relative concentrations at zero field. As shown in Figure 2a, protons lying on water–water H-bonds statistically explore the same locations independently from the relative molar ratio of water to ammonia in the mixture. By contrast, albeit up to a water concentration of 25% the protonic behavior in the H-bonds donated by a H_2_O molecule to a nearby NH_3_ one remains substantially unaltered (Figure 2b), at larger water contents such a scenario drastically changes. In fact, as displayed in Figure 2b, protons are sizably more attracted to ammonia molecules when the NH_3_ content decreases. In other words, the increment of the water content statistically pushes the protons on water-ammonia H-bonds toward the NH_3_ moiety. The population of H-bonds exhibiting large values of δ_*OwN*_, indeed, gets richer as the amount of water increases. Thus, in net contraposition to what is commonly accepted, reaction (1) tends to be shifted to the right, from an agnostic molecular perspective, not when the abundance of ammonia prevails over that of water in the mixture but exactly under the reverse situation.

**Figure 2:**
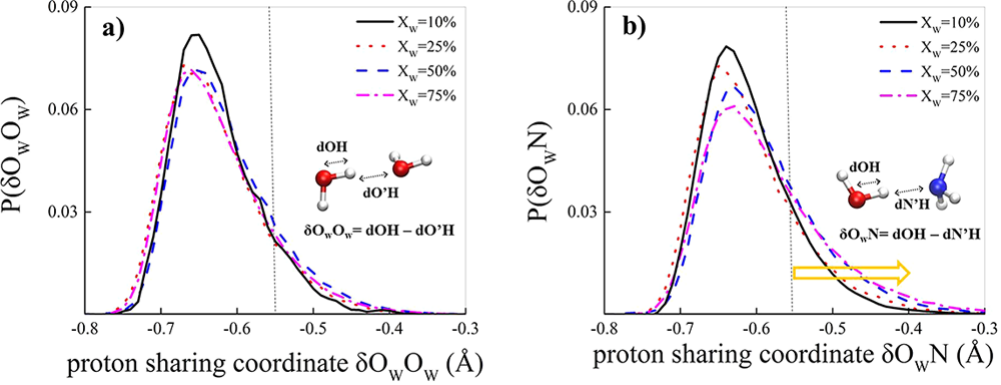
Proton sharing in H-bonds. Probability distributions of the proton sharing coordinate δ in water–water (a) and water–ammonia (b) H-bonds for 10:90 (solid black curve), 25:75 (dotted red curve), 50:50 (dashed blue curve), and 75:25 (dashed-dotted magenta curve) water–ammonia molar ratios. A vertical dotted line at δ = −0.55 Å is displayed to mark pronounced proton excursion events. In the insets, the definition of the coordinate, which is determined for every hydrogen atom involved in a tight H-bond (i.e.,δ ≥ – 0.8 Å), is shown.

To further investigate the molecular mechanisms triggering the concentration-dependent increase in reactivity of ammonia aqueous solutions, we have determined the number of first-neighbor molecules during pronounced proton excursion events along OH···N water–ammonia H-bonds (i.e., δ ≥ – 0.5 Å). These events indeed generally lead to the transient formation of the well-known NH_4_OH molecular complex.

As shown in Figure 3a, specific patterns emerge when investigating the collective behavior of the solvation shells around reactive H_2_O molecules that are prone to transiently donating H^+^ to a nearby ammonia species. In fact, independently from the composition of the mixture, a “magic” (redundant) number of first-neighbors molecular species clearly appears: ∼5, including the proton-accepting NH_3_ molecule. In other words, the proton migration mechanism in ammonia aqueous solutions is assisted by 4 generic molecules that have to simultaneously surround the forming NH_4_OH complex. Thus, regardless of the nature of the molecules in the local environment of a H_2_O species involved in an ephemeral transient proton transfer event toward a NH_3_ moiety, the cooperation between a fixed quantity (i.e., 4) of solvating molecules favors charge transfer and the instantaneous formation of the NH_4_OH molecular complex. Typical molecular arrangements observed during events associated with a large δ_*OwN*_ value are displayed in Figure 3b–e. It is worth mentioning that the hypercoordination state of the central H_2_O molecule, which is evolving into a OH^–^, resembles that observed for hydroxide ions in neat water.^44^

**Figure 3:**
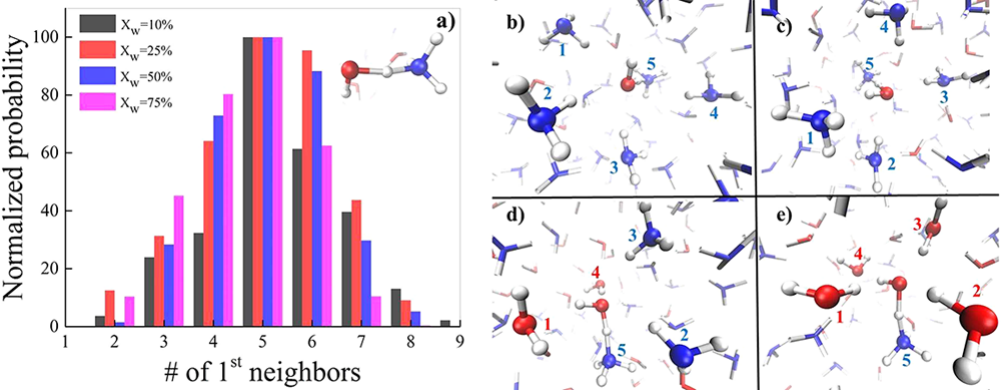
(a) Histograms showing the normalized probability of finding a given number of first-neighbor species during pronounced proton excursion events (δ ≥ – 0.5 Å), eventually leading to the formation of the NH_4_OH molecular complex (inset) and for different water/ammonia relative molar ratios (see legend for the water percentage content). (b–e) Snapshots taken from our AIMD trajectories displaying the first solvation shell around a water molecule during an ephemeral transient proton transfer event for the 10:90 (b), 25:75 (c), 50:50 (d), and 75:25 (e) water–ammonia solutions.

In light of these findings, how are peculiar molecular mechanisms capable of maximizing the macroscopic reactivity of water–ammonia solutions as a function of the relative concentration of H_2_O and NH_3_? To answer this question of paramount concern, it is worth investigating the behavior of the radial distribution functions (RDFs) *g*(*r*) and of the related running coordination numbers *n*(*r*), which allows for sketching out the solvation scenario. As shown in Figure 4a, whereas the number of water molecules surrounding a given H_2_O species progressively increases at larger water concentrations, a slight reduction of the location of the first oxygen–oxygen RDF minimum testifies to a modest contraction of the first hydration shell around H_2_O molecules. Therefore, upon adding water in ammonia aqueous solutions, H_2_O molecules become surrounded by a progressively larger amount of molecules of the same like and that lie slightly closer to each other. Conversely, the number of NH_3_ species in the proximity of H_2_O molecules decreases upon water inclusion, as displayed in Figure 4b. At the same time, the distribution of the relative H_2_O–NH_3_ distances gets significantly broader, as witnessed by the blue squares of Figure 4b, which reports the location of the first dip of the oxygen–nitrogen RDF as a function of the relative water–ammonia molar ratio (see also Figure S9 of the Supporting Information). At the highest H_2_O concentration here investigated (i.e., 75%), the first solvation shell of each water molecule is on average composed of ∼3.5 H_2_O molecules located within a radius of ∼3.4 Å (Figure 4-a) whereas ∼5 NH_3_ molecules can be found inside a bigger region of radius equal to ∼5.5 Å (Figure 4b). As a consequence, in the 75:25 water–ammonia sample, a randomly chosen water molecule directly interacts with these ∼3.5 H_2_O hydration molecules and with a portion of the ∼5 NH_3_ surrounding species.

**Figure 4:**
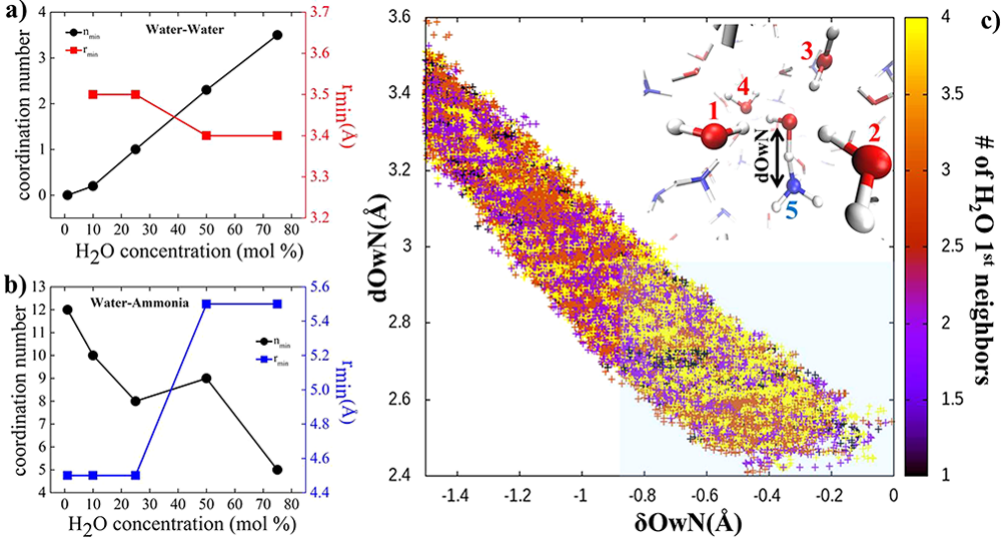
(a and b) Oxygen–oxygen and the oxygen–nitrogen coordination numbers (black circles), respectively, determined as the integral of their own RDFs up to the first minimum and the location of this latter dip (red squares, red right-handed ordinate axis for the OO and blue squares, blue right-handed ordinate for the ON) as a function of the water molar concentration. (c) Distances between H-bonded water and ammonia molecules *d*_O_*_w_*_N_ as a function of the proton sharing coordinate δ_O*w*N_, put in relationship with the number of first-neighboring H_2_O moieties in a 75:25 water–ammonia mixture. The shaded region highlights the most reactive events, one of which is visualized in the inset.

Interestingly, starting from water concentrations of 50%, the oxygen–nitrogen RDF exhibits the onset of a structured peak at 2.76 Å, which becomes more resolved in the 75:25 water–ammonia solution (Figure S9 of the Supporting Information) and whose associated coordination number is ∼1. This implies that among all the possible configurations, a water molecule in a 75:25 water–ammonia mixture finds, at typical H-bond distances, ∼3.5 H_2_O and ∼1 NH_3_ molecules. In light of the previously presented molecular pattern (Figure 3), such an average molecular arrangement, accounting for a total number of closely located first neighbors equal to ∼4.5, is very favorable for charge transfer. In fact, by mapping all distances between H-bonded water and ammonia molecules *d*_O_*_w_*_N_ with respect to the proton sharing coordinate δ_O*w*N_ and the number of first-neighbor waters, a clear microscopic scenario emerges. As displayed in Figure 4c, molecular events associated with tight H-bond formation (short *d*_O_*_w_*_N_) and that give rise to proton sharing (large δ_O*w*N_) are statistically characterized by a number of assisting water molecules equal to 4, a circumstance easily met in the 75:25 water–ammonia solution where 3.5 first-neighbor H_2_O moieties are constantly available. In other words, the most successful protolysis attempts are those maximizing the number of first-neighboring water molecules in a sort of cage effect, as displayed in the shaded region of Figure 4c. By contrast, the first solvation shell of water molecules in the remainder water–ammonia mixtures is composed by a lower number of first-neighbor H_2_O and a larger number of NH_3_ species, which, however, span larger relative distances. Such a circumstance sizably limits the reactivity-enhancing role of the water cage effect during proton transfers.

The previous analysis suggests that a H_2_O molecule finding ∼4 hydrating water species and ∼1 proton-accepting ammonia molecule lies in a more polarized state, presumably producing larger local electric fields and, hence, being more prone to donate a proton to the nearby NH_3_. To quantify the effects of the cooperative role of the solvating water molecules, we have determined the local electric field along the H-bond in proximity of the proton-accepting nitrogen atom of the ammonia species. As shown in Figure 5a, the spontaneous electric field projected onto the H-bond direction and experienced by an ammonia molecule in the H_2_O–NH_3_ dimer is 0.98 V/Å. On the other hand, the same local field is enhanced of ∼40% (i.e., 1.36 V/Å) when the coordinated cluster of molecules emerging from our simulations is considered, as displayed in Figure 5b. This finding is consistent and further corroborates recent literature of Zare’s group pointing out that the surfaces of water microdroplets can accelerate different kinds of chemical reactions owing to the presence of strong local electric fields.^19,45^

**Figure 5:**
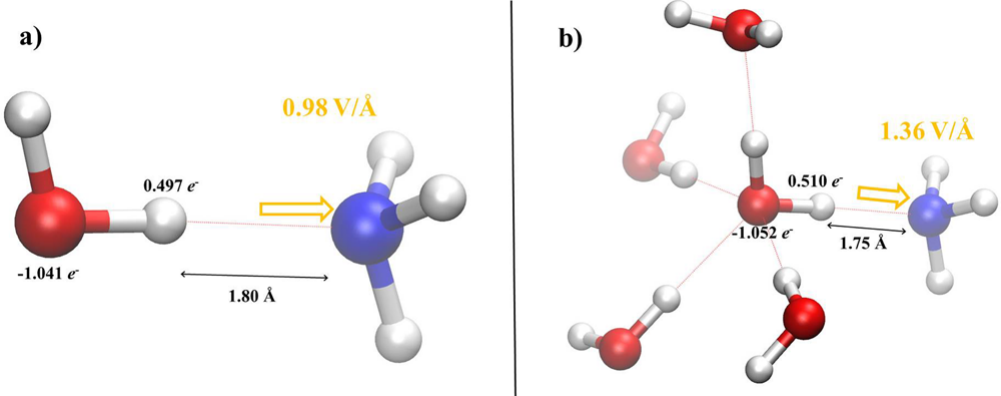
Local electric field (in yellow) spontaneously present along the H-bond and acting on the nitrogen atom of the water–ammonia dimer (a) and of the 5H_2_O–NH_3_ molecular complex (b). Most relevant atomic charges and H-bond lengths are shown for the sake of completeness.

It is worth stressing the fact that the latter finding holds also in bulk conditions and not only at the interface, as shown in Figure 6. By reprocessing 1000 randomly chosen molecular configurations taken from our AIMD trajectories at zero field, it turns out that ammonia molecules in bulk liquid ammonia experience, on average, a local electric field of 0.6 V/Å whereas a water molecule fully solvated in liquid ammonia is subjected to an average electric field strength of 1.0 V/Å (i.e., 40% larger), with peak intensities of ∼1.8 V/Å. Interestingly, stronger fields associated with the presence of water generate, in turn, more intense fields in the surrounding molecules. In fact, as displayed in Figure S11 of the Supporting Information, whereas a non-H-bonded ammonia molecule in the first solvation shell of water is subjected to an average field of 0.7 V/Å (i.e., ∼ 18% higher than its bulk counterpart), an NH_3_ species which is H-bonded to water experiences a field of 0.9 V/Å (i.e., ∼ 50% higher than that felt by bulk NH_3_ species), a value testifying—once again—to the strong polarization and hence the catalytic action exerted by the local presence of water molecules in ammonia. This way, the kosmotropic action of the local aqueous environment in combination with the high proton affinity of ammonia gives rise to the goldilocks conditions capable of enhancing molecular reactivity via the formation of stronger local electric fields.

**Figure 6:**
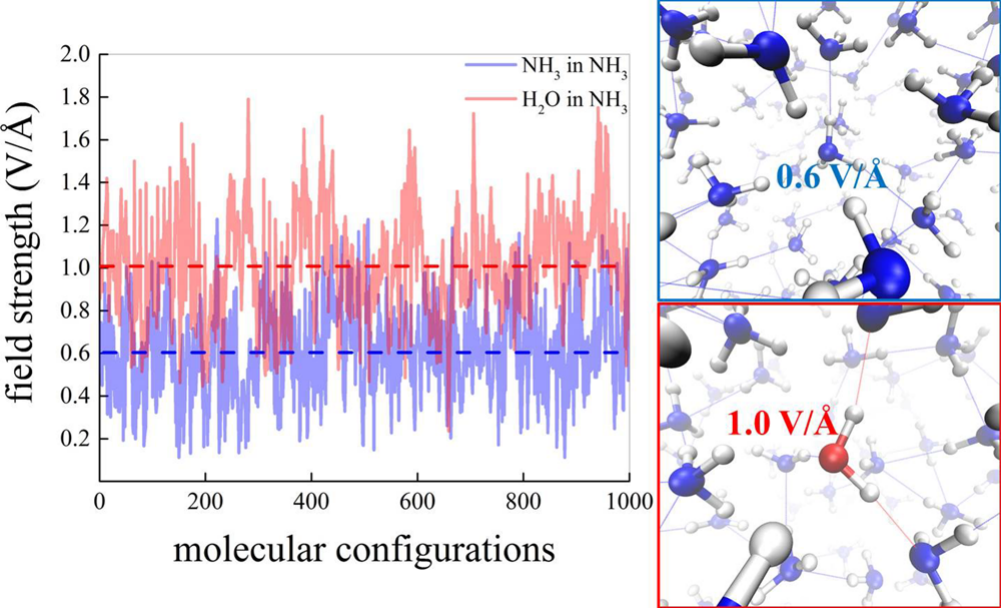
Local electric field intensity experienced by the nitrogen atom of a randomly chosen ammonia molecule in bulk liquid ammonia (blue) and by the oxygen atom of a water molecule solvated in ammonia (red). The electric-field contribution stemming from the covalently bound hydrogen atoms has been removed for genuinely tracking the effects of the liquid environment only.

Finally, in light of the molecular pattern here unveiled, where 5H_2_O molecules concertedly cooperate with 1NH_3_ species for maximizing the probability for successful charge transfers, we forecast that stoichiometric compositions satisfying such a ratio are those enhancing the acid–base behavior of the mixture (i.e., ∼ 83:17 water–ammonia). All of these observations indicate that acid and base concepts cannot be reductionistically mapped onto single-molecule features, but rather they depend on highly orchestrated dynamical phenomena emerging at larger (e.g., H-bonding) length-scales that involve strong local fields.

## Supporting Information Available

Additional data including the proton sharing coordinate distributions at different electric field intensities, radial distribution functions for diverse field strengths, local electric fields associated with H-bonding, inherent structure energy during proton transfer events, and other analyses at zero field (PDF)
