# Supplementary material for: The Reactivity-Enhancing Role of Water Clusters in Ammonia Aqueous Solutions
Source: J Phys Chem Lett. 2023 Aug 25;14(35):7808–13. doi: 10.1021/acs.jpclett.3c01810 (PMC10494223; doi:10.1021/acs.jpclett.3c01810)
Supplement: Supplementary file 1 — jz3c01810_si_001.pdf [file jz3c01810_si_001.pdf]

Supporting Information

The Reactivity-Enhancing Role of Water Clusters in Ammonia Aqueous Solutions

Giuseppe Cassone,[*,†] Franz Saija,[†] Jiri Sponer,[‡] and Sason Shaik[*,¶]

†*Institute for Physical-Chemical Processes, Italian National Research Council (IPCF-CNR), Viale Stagno d'Alcontres 37, 98158 Messina, Italy*

‡*Institute of Biophysics of the Czech Academy of Sciences, Královopolská 135, 61265 Brno (Czechia)*

¶*Institute of Chemistry, The Hebrew University of Jerusalem, Edmond J. Safra Campus, Givat Ram, Jerusalem 9190401, Israel*

E-mail: cassone@ipcf.cnr.it; sason@yfaat.ch.huji.ac.il

Methods

We used the software package CP2K,[1] based on the Born-Oppenheimer approach, to perform *ab initio* molecular dynamics (AIMD) simulations of five samples at different relative water-ammonia molar ratios (*i.e.*, 0.8:99.2, 10:90, 25:75, 50:50, 75:25) under the action of static and homogeneous electric fields applied along a given direction (corresponding to the z-axis). AIMD simulations do not employ specific parametrizations for mimicking the interactions between atoms or molecules but explicitly solve the electronic problem within a rigorous Density Functional Theory (DFT) framework. This way, interactions between nuclei genuinely result from the forces emerging from the potential energy surfaces of the

electronic subsystem. The implementation of an external field in numerical codes based on DFT can be achieved by exploiting the Modern Theory of Polarization and Berry's phases[2–4] (see, *e.g.*, Ref.[5]). Thanks to those seminal works, nowadays AIMD simulations under the effect of static electric fields with periodic boundary conditions are almost routinely carried out (see, *e.g.*, Ref.[6]).

The 0.8:99.2 water-ammonia sample ($X_W \sim 1\%$) contained 1 H_2O and 127 NH_3 molecules (*i.e.*, 511 atoms) arranged in a cubic cell with edge equal to 16.15 Å. The 10:90 water-ammonia mixture sample ($X_W = 10\%$) was composed of 13 H_2O and 115 NH_3 molecules (*i.e.*, 499 atoms) placed in a cubic box with edge of 16.18 Å. The 25:75 ammonia aqueous solution sample ($X_W = 25\%$) was composed of 32 water and 96 ammonia molecules (*i.e.*, 480 atoms) put in a cubic box having side equal to 16.22 Å. The 50:50 (equimolar) water-ammonia sample ($X_W = 50\%$) was composed of 64 H_2O and 64 NH_3 molecules (*i.e.*, 448 atoms) placed in a cubic box with edge equal to 16.30 Å. Finally, the 75:25 water-ammonia mixture sample ($X_W = 75\%$) was composed of 96 H_2O and 32 NH_3 molecules (*i.e.*, 416 atoms) arranged in a cubic box having edge of 16.38 Å. As usual, in order to minimize nonphysical surface and finite-size effects, all structures were replicated in space by employing periodic boundary conditions. The intensity of the electric field was gradually increased with a step increment of 0.05 V/Å from zero up to a maximum of 0.50 V/Å to intercept the molecular dissociation thresholds in all samples. In the zero-field cases we performed dynamics of 20 ps, whereas for each other value of the field intensity we ran dynamics of 10 ps. A time-step of 0.5 fs has been chosen.

Wavefunctions of the atomic species have been expanded in triple-zeta valence plus polarization (TZVP) basis sets with Goedecker-Teter-Hutter pseudopotentials using the Gaussian plane waves (GPW) method.[7] A plane-wave cutoff of 400 Ry has been imposed. Exchange and correlation (XC) effects were treated with the Becke-Lee-Yang-Parr (BLYP)[8,9] density functional. Moreover, in order to take into account dispersion interactions, we employed the dispersion-corrected version of BLYP (*i.e.*, BLYP-D3).[10,11] Adoption of the BLYP-D3

functional has been encouraged not only by the evidence that such a functional – when dispersion corrections are taken into account – offers one of the best adherence with the experimental results for H-bonded systems among the Generalized Gradient Approximation (GGA) XC functionals,[12,13] but also by the fact that its pure version (BLYP) already adequately works for neat liquid ammonia.[14] Furthermore, recent AIMD simulations of pure ammonia[15] at the BLYP+D3/TZVP level have shown that radial distribution functions, infrared spectral features, and molecular diffusion coefficients are all in fairly good agreement with experiments.[16–18] The dynamics of nuclei was simulated classically within a constant number, volume, and temperature (NVT) ensemble, using the Verlet algorithm whereas the canonical sampling has been executed by employing a canonical-sampling-through-velocity-rescaling thermostat[19] set with a time constant equal to 20 fs. Simulations were carried out at the nominal temperature of 295 K.

Evaluation of local electric fields on selected sub-structures extracted from the AIMD trajectories was performed by re-optimizing the molecular geometries at the B3LYP/6-311++G(d,p) DFT level under implicit water solvation (SMD) and by exploiting the TITAN code.[20] The same code was also used to determine local electric fields in liquid simulation boxes of neat ammonia and in the 0.8:99.2 water-ammonia mixtures by extracting and re-processing 1000 randomly chosen snapshots from the original AIMD trajectories.

Additional results

Electric-field-induced molecular dissociation thresholds triggering protolysis events in all systems have been determined by means of a series of quantities, including the proton sharing coordinate δ. As laid out in the main text, this indicator is capable of monitoring proton excursion events in the H-bond network and is defined as $\delta = dOH - dX'H$, where dOH is the covalent bond length of a reference H_2O molecule, whereas $dX'H$ – with $X = O, N$ – represents the length of the H-bond(s) that such a reference molecule donates – either to

a nearby H_2O or NH_3 species – as depicted in the insets of Fig. 2 of the main text. Since, of course, at all the concentrations explored in the current investigation the first ionization events stem from the dissociation of a water molecule toward an ammonia one, the here presented evaluation refers to the proton sharing coordinate $\delta_{OwN} = dOH - dN'H$ only (see the inset of Fig. 2-b of the main text for a schematic visualization).

Generally speaking, such a coordinate provides an as much as possible agnostic evaluation of proton transfer events. In fact, tails of the logarithm of the probability distributions of the proton sharing coordinate δ_{OwN} are able to sharply separate statistical fluctuations of thermal and/or chemical nature of the OH bond length from net field-induced molecular dissociations. A useful criterion for identifying field-driven proton migrations is represented by the probability of finding a dissociated molecule greater than 10^{-4}, as reported elsewhere.[21] Such a condition (*i.e.*, $P(\delta \rightarrow 0) > 10^{-4}$) is fulfilled at 0.30 V/Å (orange curve in Fig. S1-a), a

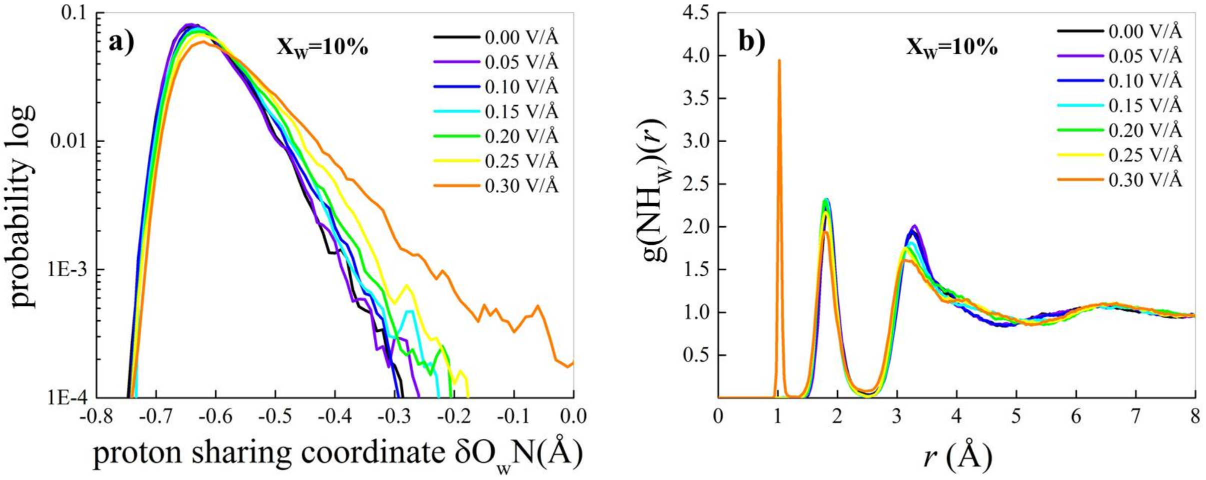

Figure S1: (a) Probability distribution in logarithmic scale of the proton sharing coordinate in the network of H-bonds donated by waters and accepted by ammonia molecules in the 10:90 water-ammonia mixture at different field strengths (see legend). (b) Intermolecular radial distribution function (RDF) between the nitrogen atoms of ammonia and the hydrogen atoms of water in the 10:90 water-ammonia mixture at different field strengths (see legend). The onset of a new intramolecular peak at ~ 1 Å at a field intensity of 0.30 V/Å testifies successful proton transfer event(s).

field value marking the ionization threshold of the water-ammonia mixture when the relative molar ratio of its constituents is equal to 10:90 (*i.e.*, $X_W = 10\%$). Additionally to previous investigations for bulk liquid water,[21] such a criterion has demonstrated to be reliable also

in ammonia aqueous samples, *a posteriori*, by comparison of the probability distributions of δ_{OwN} with on-the-fly atomistic radial distribution functions (RDFs), direct inspection of the trajectories, and the monitoring of ionic species formation (see, *e.g.*, Fig. 3-b/e of the main text). In fact, the intermolecular RDF between the nitrogen atoms and the water hydrogen atoms exhibits a novel intramolecular peak located at about 1 Å as a consequence of proton exchange(s) event(s) at a ionization threshold of 0.30 V/Å in the 10:90 water-ammonia mixture, as displayed in Fig. S1-b.

A similar joint analysis allowed us to collocate the minimum field-threshold for protolysis in all the remainder systems, as shown in Fig. S2, S3, and S4 for the 25:75, 50:50, and 75:25 water-ammonia samples, respectively. As for the 25:75 water-ammonia mixture, the

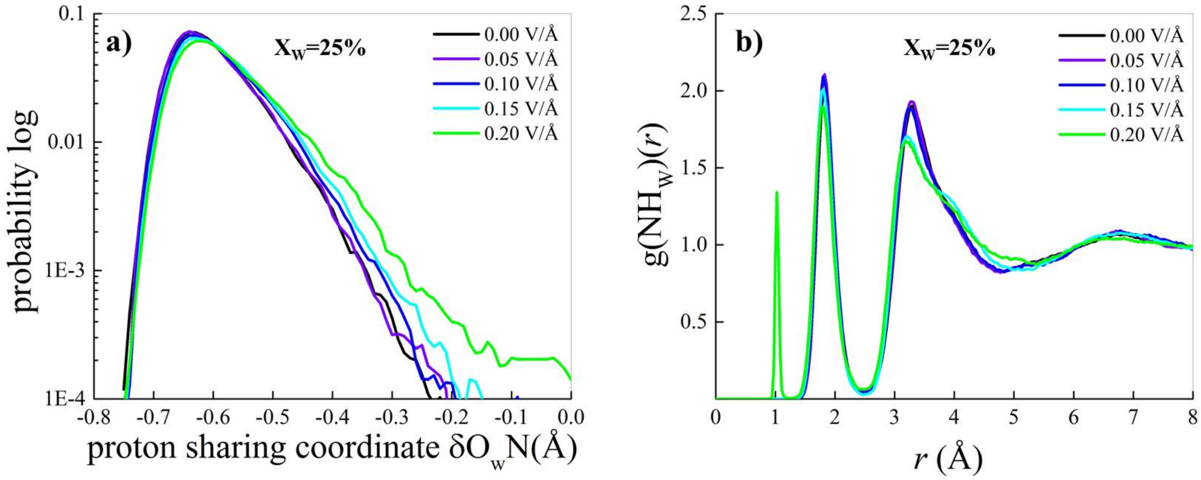

Figure S2: (a) Probability distribution in logarithmic scale of the proton sharing coordinate in the network of H-bonds donated by waters and accepted by ammonia molecules in the 25:75 water-ammonia mixture at different field strengths (see legend). (b) Intermolecular radial distribution function (RDF) between the nitrogen atoms of ammonia and the hydrogen atoms of water in the 25:75 water-ammonia mixture at different field strengths (see legend). The onset of a new intramolecular peak at ~ 1 Å at a field intensity of 0.20 V/Å testifies successful proton transfer event(s).

first ionization events are recorded for a field threshold of 0.20 V/Å, as witnessed by the tail of the logarithmic probability distribution of δ_{OwN} at the latter field intensity (green curve of Fig. S2-a). Under those circumstances, a $P(\delta \to 0) > 10^{-4}$ is observed, jointly with the evidence that also the intermolecular RDF between the nitrogen atoms and the water hydrogens exhibits the birth of a novel intramolecular peak, as visible from Fig. S2-b.

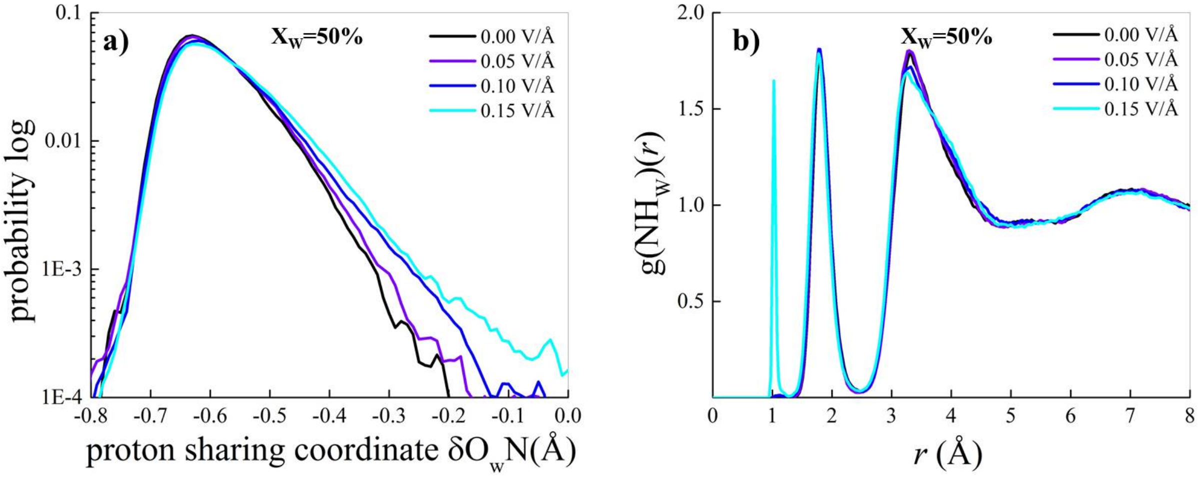

Figure S3: (a) Probability distribution in logarithmic scale of the proton sharing coordinate in the network of H-bonds donated by waters and accepted by ammonia molecules in the equimolar 50:50 water-ammonia mixture at different field strengths (see legend). (b) Intermolecular radial distribution function (RDF) between the nitrogen atoms of ammonia and the hydrogen atoms of water in the equimolar 50:50 water-ammonia mixture at different field strengths (see legend). The onset of a new intramolecular peak at ~ 1 Å at a field intensity of 0.15 V/Å testifies successful proton transfer event(s).

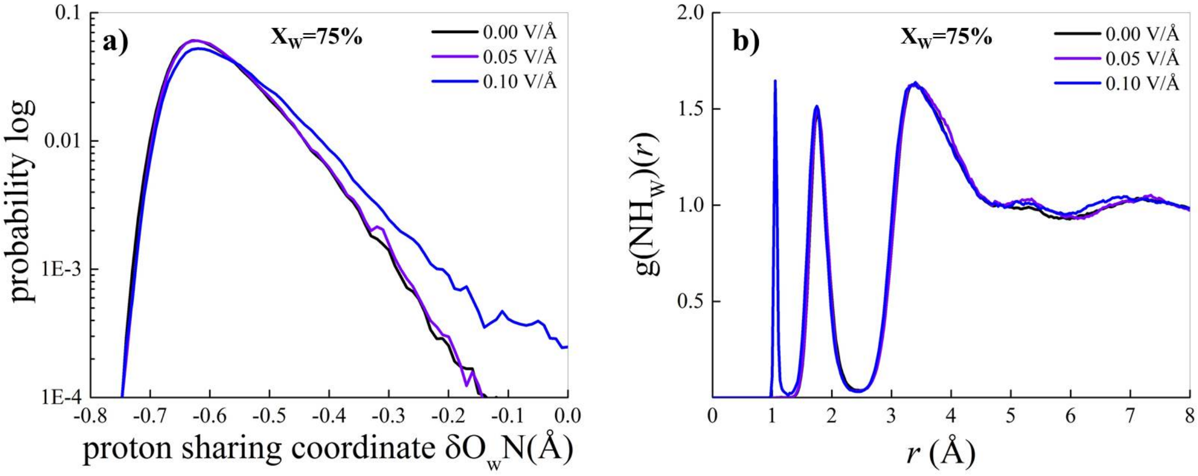

Figure S4: (a) Probability distribution in logarithmic scale of the proton sharing coordinate in the network of H-bonds donated by waters and accepted by ammonia molecules in the 75:25 water-ammonia mixture at different field strengths (see legend). (b) Intermolecular radial distribution function (RDF) between the nitrogen atoms of ammonia and the hydrogen atoms of water in the 75:25 water-ammonia mixture at different field strengths (see legend). The onset of a new intramolecular peak at ~ 1 Å at a field intensity of 0.10 V/Å testifies successful proton transfer event(s).

Besides, a similar chemical scenario – testifying a sizably larger reactivity of the protonic subsystem – is recorded at lower field strengths for the equimolar and the 75:25 water-ammonia samples. In fact, as shown in Fig. S3, water molecules donate protons to the ammonia ones already at intensities of 0.15 V/Å in the 50:50 solution. Such a field threshold is further lowered to 0.10 V/Å when an excess of water is present in the 75:25 water-ammonia mixture, as displayed in Fig. S4.

An equivalent analysis has been conducted for the 0.8:99.2 water-ammonia sample (*i.e.*, $X_W = 0.8\%$). Because of an intrinsically poorer statistics due to the employment of a single water molecule solvated by 127 ammonia species, the statistical noise at large values of δ_{OwN} is markedly higher than in all previous cases, as shown in Fig. S5-a. On the other hand, the simulation *via* AIMD methods and in presence of external electric fields of boxes reproducing such a relatively extreme molar ratio represents a sort of computational upper bound, accounting to a total number of simulated atoms equal to 511, as mentioned in the Methods section of the main text. Moreover, by crossing the data stemming from the proton

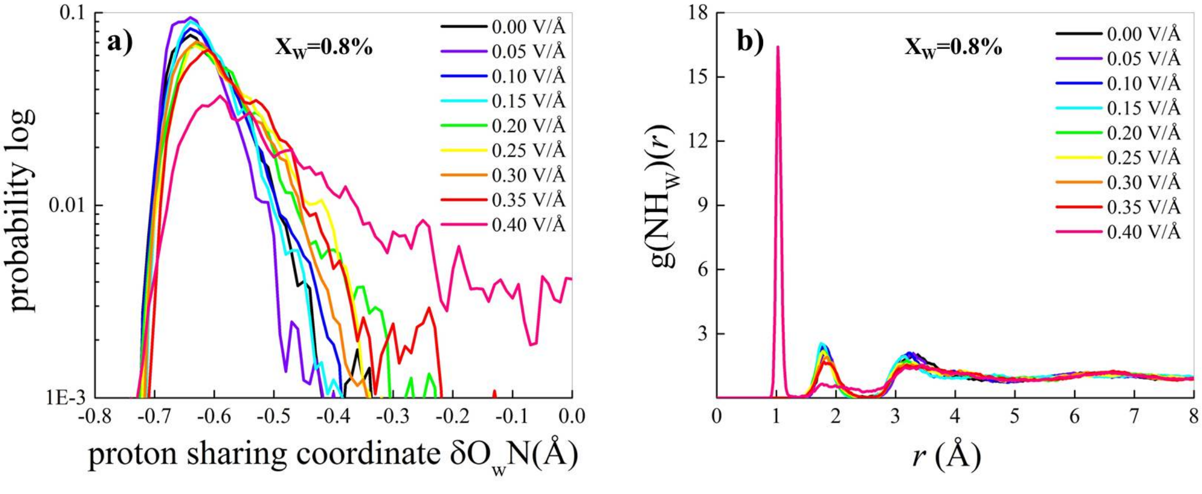

Figure S5: (a) Probability distribution in logarithmic scale of the proton sharing coordinate in the network of H-bonds donated by waters and accepted by ammonia molecules in the 0.8:99.2 water-ammonia mixture at different field strengths (see legend). (b) Intermolecular radial distribution function (RDF) between the nitrogen atoms of ammonia and the hydrogen atoms of water in the 0.8:99.2 water-ammonia mixture at different field strengths (see legend). The onset of a new intramolecular peak at ~ 1 Å at a field intensity of 0.40 V/Å testifies successful proton transfer event(s).

sharing coordinate with those emerging from the intermolecular RDF between the nitrogen

atoms and the water hydrogens, an unambiguous electric-field-induced water dissociation threshold appears. As shown in Fig. S5-b, indeed, the onset of the pronounced intramolecular peak at 0.40 V/Å marks the occurrence of proton transfers. This finding is not obvious in light of the evidence that electric field strengths of double magnitude (*i.e.*, $\sim$ 0.8 V/Å) are not capable of dissociating molecules in neat ammonia samples, as reported in Ref.;[15] the inclusion of small amounts of water, hence, awakens a silent reactivity in ammonia, a circumstance magnifying the catalytic role of water molecules.

In Fig. 2-b of the main text, it is shown that protons are sizably more attracted by ammonia molecules when the content of ammonia relative to that of water decreases. In other words, the increment of the water content statistically pushes the protons on water-ammonia H-bonds toward the NH_3 moiety. The population of H-bonds exhibiting large values of δ_{OwN} becomes indeed richer as the amount of water increases. Although one might be tempted to interpret this counterintuitive result as a genuine statistical effect introduced by the fact that larger amount of water may create a larger number of $HOH\cdots NH_3$ pairs and related proton excursion events, this is manifestly disproved by the fact that also the corresponding proton excursions on $HOH\cdots H_2O$ bonds should be affected by a similar modification. However, as shown in Fig. 2-a of the main text, no change is recorded in the distribution $P(\delta_{OwOw})$ as a function of the relative concentration of water and ammonia. In addition, the quality of the proton sharing coordinate is further testified by the fact that the well-known higher proton affinity of NH_3 molecules is perfectly caught at standard conditions (*i.e.*, at zero field) by the distributions of δ_{OwN} when compared to their counterparts δ_{OwOw} for all the investigated samples, as shown in Fig. S6. Fig. S7 shows that by introducing ammonia in water, a slight reduction of the tendency toward water-to-water proton transfers is recorded for all the relative water-ammonia concentrations here investigated. Not surprisingly, indeed, the larger the amount of ammonia the smaller the tendency of the systems toward proton migrations originating from a H_2O molecule and ending into another H_2O species. Conversely, as shown in Fig. S7, the propensity of sharing protons from a water species to an ammonia one is

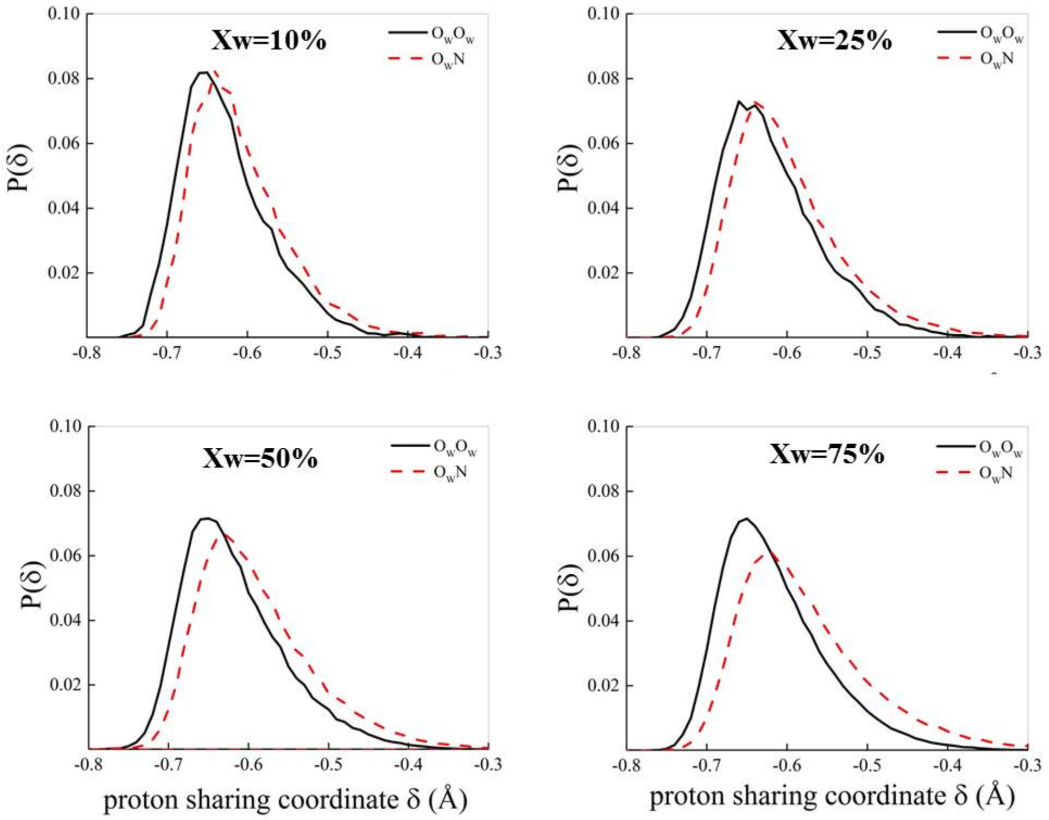

Figure S6: Probability distributions of the proton sharing coordinates in the networks of H-bonds donated by water molecules and accepted by water molecules (solid black curves) and in that formed by H-bond donating water species and H-bond accepting ammonia molecules (dashed red curves) in water-ammonia mixtures with different relative molar ratio and at standard conditions (*i.e.*, at zero field). Water content with respect to the total molar amount is reported as figure title.

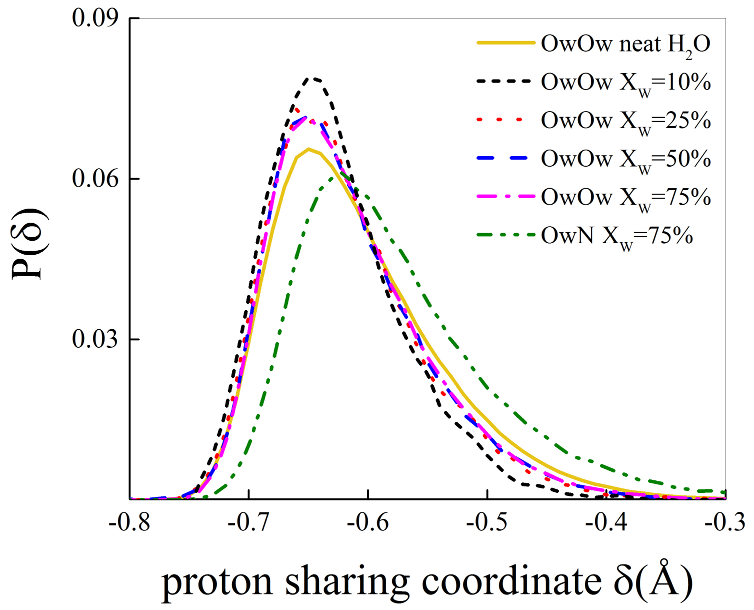

Figure S7: Probability distribution of the proton sharing coordinate δ_{OwOw} between water molecules in bulk water (solid yellow curve) and in water-ammonia mixtures at different relative concentrations (see legend reporting the percent molar fraction of the water content). For comparison, also the proton sharing coordinate δ_{OwN} between H-bond donating water species and H-bond accepting ammonia molecules in the 75:25 water-ammonia mixture is reported (dashed-dotted-dotted green curve).

measurably larger in (the 75:25) water-ammonia mixture(s) (dashed-dotted-dotted green curve) with respect to the neat water case (solid yellow curve); this evidence quantitatively testifies the higher proton affinity of ammonia molecules *vis-à-vis* water molecules.

In Fig. S8 and S9 the oxygen-oxygen and oxygen-nitrogen RDFs at standard conditions (*i.e.*, at zero field) for different water-ammonia relative molar ratios are reported, respectively. Although modifications of the main features as a function of the relative concentration are fully consistent with those observed in a pioneering computational investigation,[22] the systems simulated in the present work not only span a wider palette of relative water-ammonia concentrations but are also significantly bigger and allow for evaluating the effects introduced by a change in composition up to the second/third solvation shell.

In Fig. S10 we underline the role played by the hydrating water molecules located in a radius of 3.5 Å in assisting proton excursion events from a H_2O species toward a NH_3 one. These events correspond to relatively short intermolecular H_2O-NH_3 distances and large δ_{OwN} values. Notwithstanding the small overall amount of water in the 10:90 (X_W = 10%) and

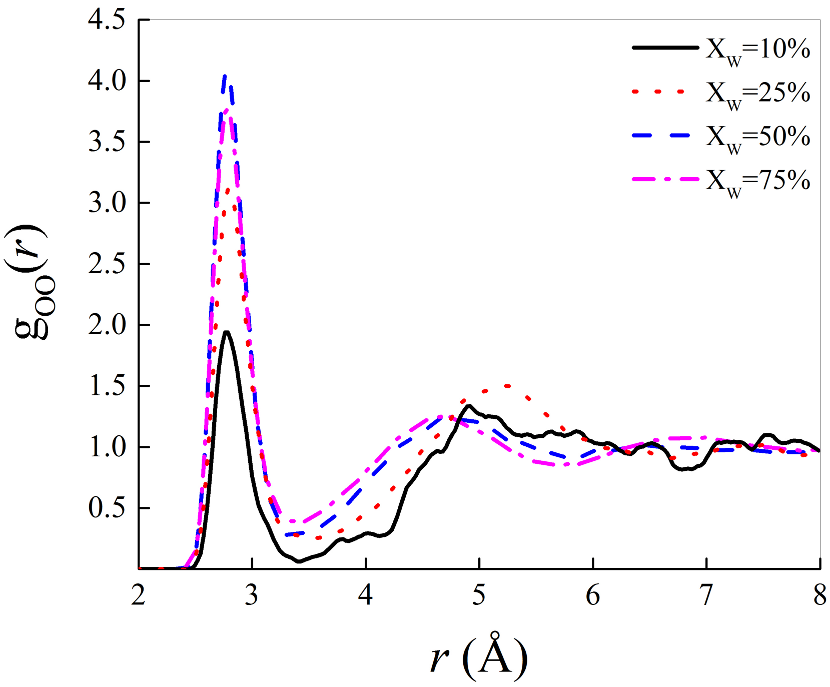

Figure S8: Oxygen-oxygen RDF in 10:90 (solid black curve), 25:75 (dotted red curve), 50:50 (dashed blue curve), and 75:25 (dashed-dotted magenta curve) water-ammonia mixtures.

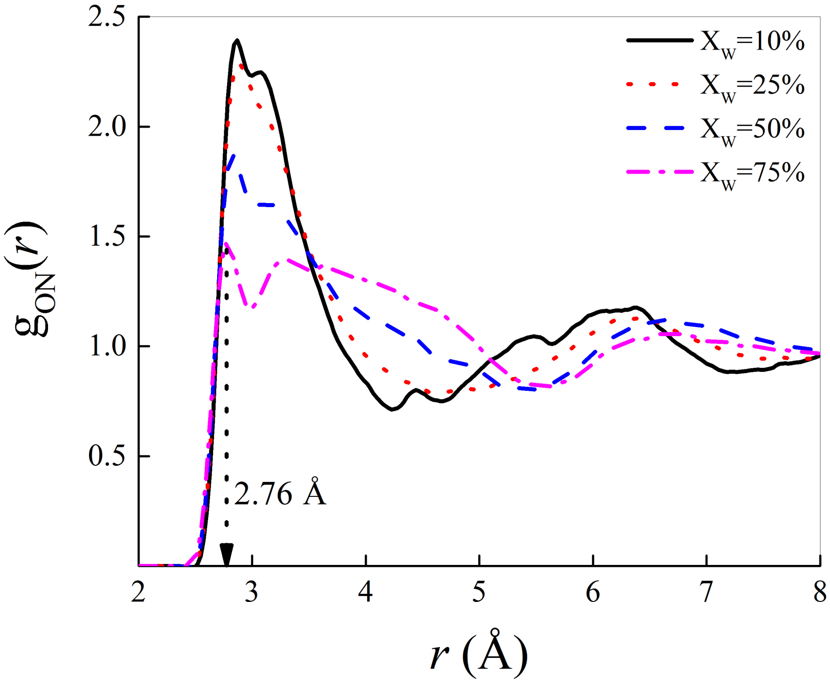

Figure S9: Oxygen-nitrogen RDF in 10:90 (solid black curve), 25:75 (dotted red curve), 50:50 (dashed blue curve), and 75:25 (dashed-dotted magenta curve) water-ammonia mixtures. The dashed arrow marks the 1st peak position at 2.76 Å.

25:75 (X_W = 25%) water-ammonia mixtures, the "most reactive events" or, equivalently, the "most reactive H-bonds" manifest themselves by means of the intercession of the maximally-available local amount of water. Of course, such a circumstance is much more evident in the samples where the water content is either equivalent (50:50, X_W = 50%) or larger (75:25, X_W = 75%) than the amount of ammonia.

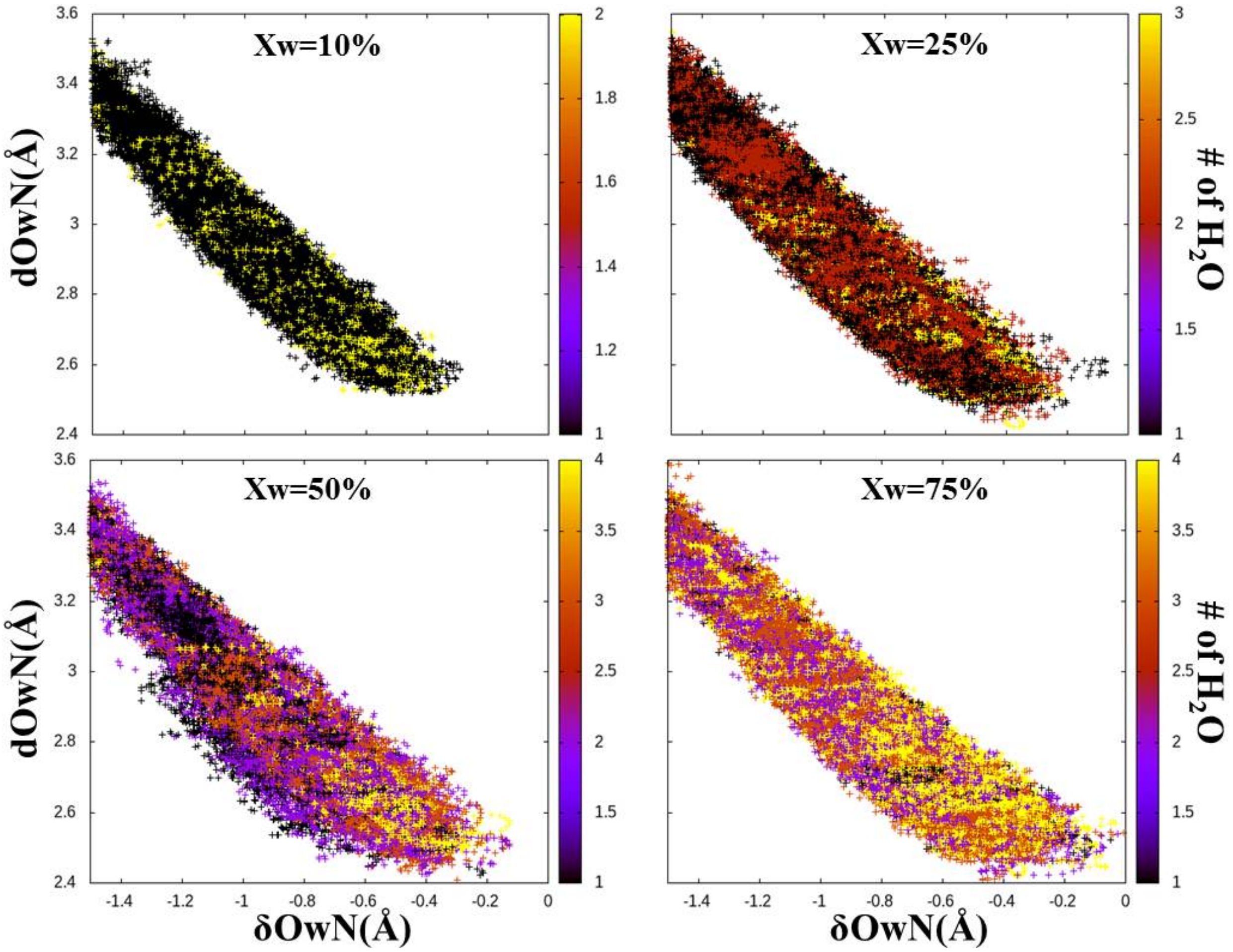

Figure S10: Distances between H-bonded water and ammonia molecules $dOwN$ as a function of the proton sharing coordinate δ_{OwN}, put in relationship with the number of first-neighboring H_2O (*i.e.*, located within a radius of 3.5 Å from the central water molecule) in water-ammonia mixtures with different relative molar ratio and at standard conditions (*i.e.*, at zero field). Water content with respect to the total molar amount is reported as figure title.

As in every computational investigation dealing with activated chemical processes, it is tempting to determine the free energy associated with the phenomenon under investigation. However, as laid out in the main text, highly cooperative mechanisms are responsible for the seminal molecular dissociation events and for the subsequent proton transfers across

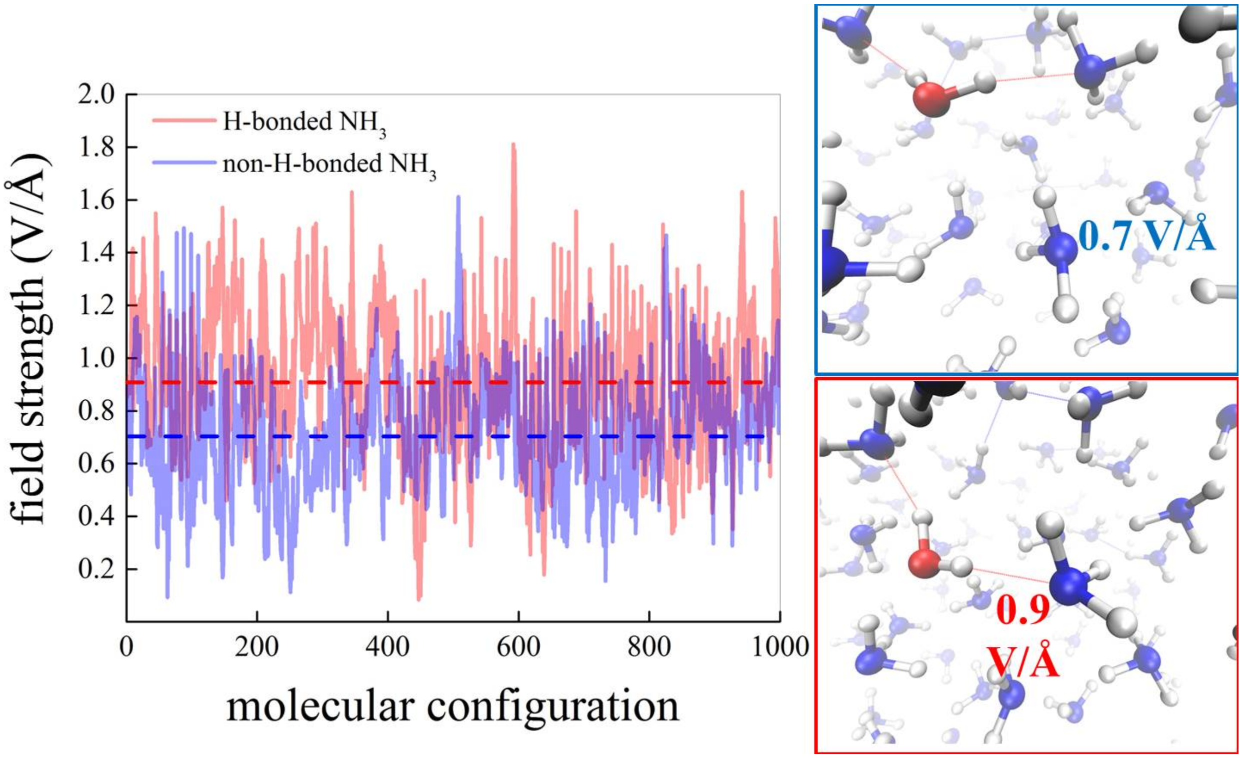

Figure S11: Local electric field intensity experienced by the nitrogen atom of a non-H-bonded ammonia molecule in the first solvation shell a water molecule (blue) and by the nitrogen atom of an ammonia molecule which is H-bonded to the water moiety (red). An average increment of about 0.20 V/Å can be associated to the establishment of the H-bond interaction under the investigated circumstances.

the H-bond networks of the various water-ammonia liquid systems. Quantum-mechanical calculations carried out under implicit solvation conditions are not capable of adequately modelling reactions where charged species are firstly synthesized and then stabilized owing to the explicit intervention of the surrounding molecular environment. Furthermore, the option of evaluating free-energy estimates *via* traditional quantum chemistry calculations is clearly discarded if one takes into account a basic lesson from general electrostatics: oppositely charged species (*e.g.*, such as the reaction products OH^- and $NH_4{}^+$) subjected to the action of an external electric field are forced to move apart by the underlying electrostatic potential gradient until they reach an infinite distance from each other, a circumstance impossible to be achieved in non-periodic quantum chemistry calculations by construction.

Another option would be that of performing prolonged AIMD simulations coupled with enhanced sampling techniques, such as, *e.g.*, metadynamics.[23] Some of us have used, in previous investigations, those techniques for complex chemical reactions occurring in the presence of external fields.[24] However, typical error bars associated with those techniques are generally on the same order of magnitude of the free-energy barrier height for molecular ionization (*i.e.*, 2−3 kcal/mol),[25] rendering their usage likely ineffective – with not-conclusive outcomes – for proton transfer phenomena. Also because of these reasons, the free energy associated with water ionization was historically investigated with other techniques involving the constraint of the degrees of freedom of the system:[26] not an optimal scenario for processes occurring in highly correlated molecular systems where cooperative dynamics has been proven to be essential (see main text).

In light of these evidences, and with the aim of explicitly including the liquid character of the system, the best option in our hands allowing for a rough estimate of the contribution carried by the external field in modifying the underlying energy landscape is that of determining the so-called inherent structure energy, which mirrors the potential energy landscape while removing the thermal noise. This way, taking advantage of the *ab initio* trajectories generated in presence of a field intensity of 0.15 V/Å in the 50 : 50 water-ammonia sample,

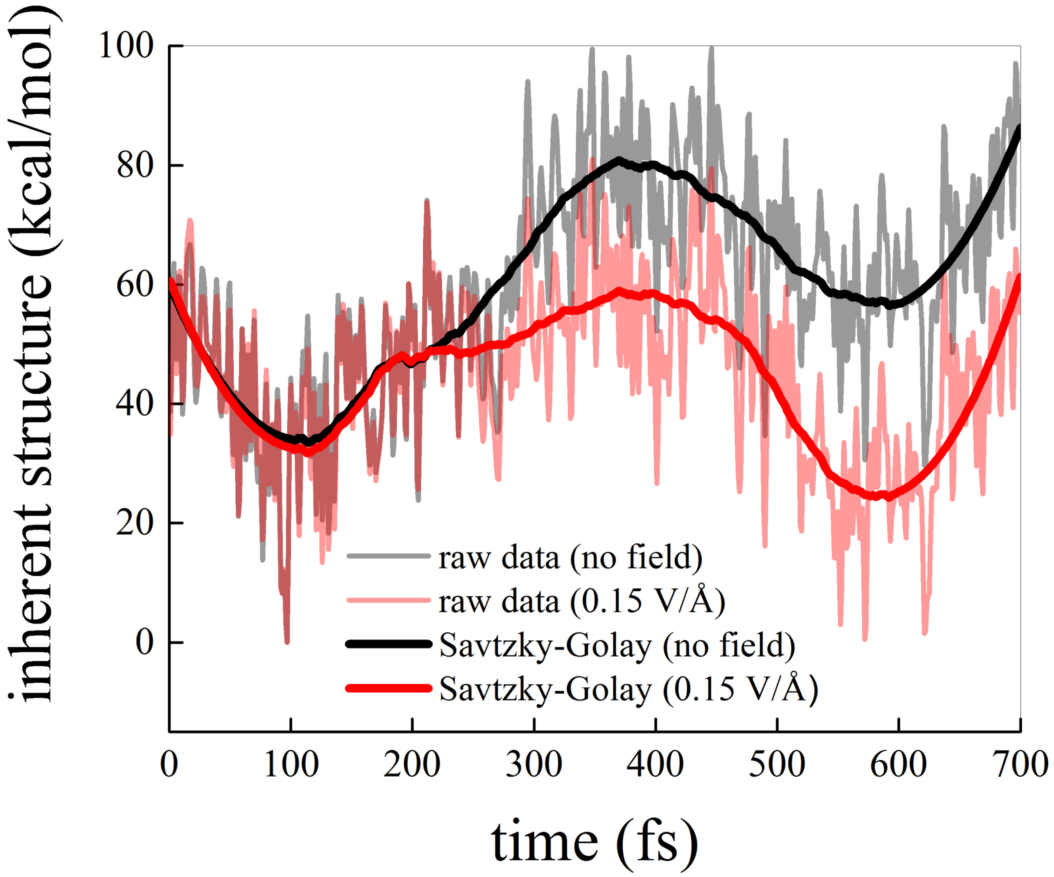

Figure S12: Inherent structure energy associated with a typical field-induced proton transfer event in the equimolar water-ammonia mixture. Large fluctuations of the potential energy landscape are continuously recorded in simulation boxes of liquid systems. A Savitzky-Golay smoothing better magnifies the existence of local minima associated with the reactants and products states (see also Fig. S13).

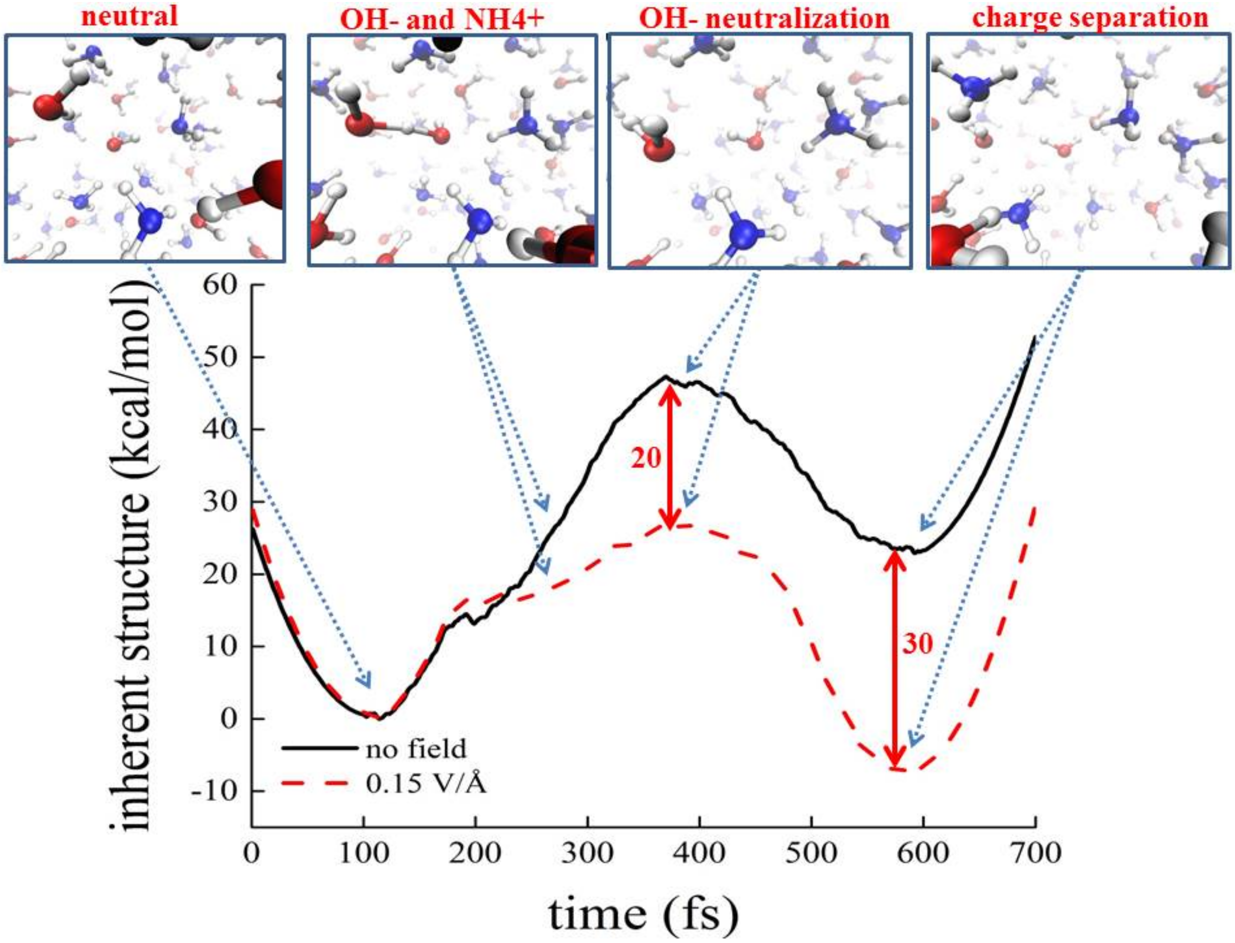

Figure S13: Savitzky-Golay smoothing of the potential energy landscape of Fig. S12 rescaled to the energy of the reactants basin. The presence of the external electric field flattens the portion of the landscape associated with the initial charged pair (OH^- and $NH_4{}^+$) formation. Moreover, the location of the saddle point identifying the putative "transition state" (notice that this is not the free energy!) of the proton transfer process is lowered in energy by about 20 kcal/mol. Finally, one of the main effects carried by the field is associated with the manifest stabilization (*i.e.*, by $\sim$ 30 kcal/mol) of the products basin, where charged species are formed and separated by the field in the liquid simulation box.

we reprocessed a series of spontaneous proton transfer events in presence of the latter external field and determined for that the potential energy profiles shown in Fig. S12 and S13. In particular, the inherent structure is determined by quenching to 0 K the molecular configurations stemming from the dynamics – hence removing thermal noise while keeping the exploration of the phase space – and by determining the respective instantaneous energy. In other words, we have executed a series of self-consistent field calculations (*i.e.*, single-point calculations) with and without the external field on frames extracted from AIMD trajectories in presence of the field and exhibiting proton transfer events. For this reason, the temporal evolution (*i.e.*, x-axis) in Fig. S12 and S13 appears to be unaffected by the presence of the field. On the other hand, the fact that the saddle point is lowered in energy by about 20 kcal/mol represents another evidence showing the field-induced enhancement of the kinetics of the reaction, in addition to the manifest stabilization (by $\sim$ 30 kcal/mol) of the charged reaction products in the liquid (Fig. S13). Although not capable of affording a free-energy evaluation, this strategy allows for the determination of the potential energy landscape of dynamical processes occurring in explicitly treated solvents and under periodic boundary conditions, essential aspects to be included for tracking proton transfer phenomena in condensed matter. The reader who is interested in the effects carried by the application of strong electric fields on the topology of complex free-energy surfaces can refer to Refs.[24,27]

References

(1) Kühne, T. D. et al. CP2K: An electronic structure and molecular dynamics software package - Quickstep: Efficient and accurate electronic structure calculations. *The Journal of Chemical Physics* **2020**, *152*, 194103.

(2) King-Smith, R. D.; Vanderbilt, D. Theory of polarization of crystalline solids. *Phys. Rev. B* **1993**, *47*, 1651–1654.

(3) Resta, R. Macroscopic polarization in crystalline dielectrics: the geometric phase approach. *Rev. Mod. Phys.* **1994**, *66*, 899–915.

(4) Berry, M. V. Quantal phase factors accompanying adiabatic changes. *Proceedings of the Royal Society of London. A. Mathematical and Physical Sciences* **1984**, *392*, 45–57.

(5) Umari, P.; Pasquarello, A. Ab initio Molecular Dynamics in a Finite Homogeneous Electric Field. *Phys. Rev. Lett.* **2002**, *89*, 157602.

(6) English, N. J.; Waldron, C. J. Perspectives on external electric fields in molecular simulation: progress, prospects and challenges. *Phys. Chem. Chem. Phys.* **2015**, *17*, 12407–12440.

(7) Krack, M. Pseudopotentials for H to Kr optimized for gradient-corrected exchange-correlation functionals. *Theoretical Chemistry Accounts* **2005**, *114*, 145–152.

(8) Becke, A. D. Density-functional exchange-energy approximation with correct asymptotic behavior. *Phys. Rev. A* **1988**, *38*, 3098–3100.

(9) Lee, C.; Yang, W.; Parr, R. G. Development of the Colle-Salvetti correlation-energy formula into a functional of the electron density. *Phys. Rev. B* **1988**, *37*, 785–789.

(10) Grimme, S.; Antony, J.; Ehrlich, S.; Krieg, H. A consistent and accurate ab initio parametrization of density functional dispersion correction (DFT-D) for the 94 elements H-Pu. *The Journal of Chemical Physics* **2010**, *132*, 154104.

(11) Grimme, S.; Ehrlich, S.; Goerigk, L. Effect of the damping function in dispersion corrected density functional theory. *Journal of Computational Chemistry* **2011**, *32*, 1456–1465.

(12) Lin, I.-C.; Seitsonen, A. P.; Tavernelli, I.; Rothlisberger, U. Structure and Dynamics of Liquid Water from ab Initio Molecular Dynamics—Comparison of BLYP, PBE, and revPBE Density Functionals with and without van der Waals Corrections. *Journal of Chemical Theory and Computation* **2012**, *8*, 3902–3910, PMID: 26593030.

(13) Bankura, A.; Karmakar, A.; Carnevale, V.; Chandra, A.; Klein, M. L. Structure, Dynamics, and Spectral Diffusion of Water from First-Principles Molecular Dynamics. *The Journal of Physical Chemistry C* **2014**, *118*, 29401–29411.

(14) Boese, A. D.; Chandra, A.; Martin, J. M. L.; Marx, D. From ab initio quantum chemistry to molecular dynamics: The delicate case of hydrogen bonding in ammonia. *The Journal of Chemical Physics* **2003**, *119*, 5965–5980.

(15) Cassone, G.; Sponer, J.; Sponer, J. E.; Saija, F. Electrofreezing of Liquid Ammonia. *The Journal of Physical Chemistry Letters* **2022**, *13*, 9889–9894, PMID: 36255376.

(16) Bromberg, A.; Kimel, S.; Ron, A. Infrared spectrum of liquid and crystalline ammonia. *Chemical Physics Letters* **1977**, *46*, 262–266.

(17) Zheng, W.; Kaiser, R. I. An infrared spectroscopy study of the phase transition in solid ammonia. *Chemical Physics Letters* **2007**, *440*, 229–234.

(18) O'Reilly, D. E.; Peterson, E. M.; Scheie, C. E. Self-diffusion in liquid ammonia and deuteroammonia. *The Journal of Chemical Physics* **1973**, *58*, 4072–4075.

(19) Bussi, G.; Donadio, D.; Parrinello, M. Canonical sampling through velocity rescaling. *The Journal of Chemical Physics* **2007**, *126*, 014101.

(20) Stuyver, T.; Huang, J.; Mallick, D.; Danovich, D.; Shaik, S. TITAN: A Code for Modeling and Generating Electric Fields—Features and Applications to Enzymatic Reactivity. *Journal of Computational Chemistry* **2020**, *41*, 74–82.

(21) Cassone, G. Nuclear Quantum Effects Largely Influence Molecular Dissociation and Proton Transfer in Liquid Water under an Electric Field. *The Journal of Physical Chemistry Letters* **2020**, *11*, 8983–8988, PMID: 33035059.

(22) Bankura, A.; Chandra, A. A first principles molecular dynamics study of the solvation structure and migration kinetics of an excess proton and a hydroxide ion in binary water-ammonia mixtures. *The Journal of Chemical Physics* **2012**, *136*, 114509.

(23) Laio, A.; Parrinello, M. Escaping free-energy minima. *Proceedings of the National Academy of Sciences* **2002**, *99*, 12562–12566.

(24) Cassone, G.; Pietrucci, F.; Saija, F.; Guyot, F.; Saitta, A. M. One-step electric-field driven methane and formaldehyde synthesis from liquid methanol. *Chem. Sci.* **2017**, *8*, 2329–2336.

(25) Agmon, N. The Grotthuss mechanism. *Chemical Physics Letters* **1995**, *244*, 456 – 462.

(26) Sprik, M. Computation of the pK of liquid water using coordination constraints. *Chemical Physics* **2000**, *258*, 139–150.

(27) Cassone, G.; Pietrucci, F.; Saija, F.; Saitta, A. M. In *Computational Approaches for Chemistry Under Extreme Conditions*; Goldman, N., Ed.; Springer International Publishing: Cham, 2019; pp 95–126.
